# Targeting Cell Cycle Progression in HER2+ Breast Cancer: An Emerging Treatment Opportunity

**DOI:** 10.3390/ijms23126547

**Published:** 2022-06-11

**Authors:** Nischal Koirala, Nandini Dey, Jennifer Aske, Pradip De

**Affiliations:** Translational Oncology Laboratory, Avera Cancer Institute, Sioux Falls, SD 57105, USA; nischal.koirala@avera.org (N.K.); nandini.dey@avera.org (N.D.); jennifer.aske@avera.org (J.A.)

**Keywords:** brain metastasis, cell cycle inhibitors, cell cycle pathway, ERBB2, HER2+ breast cancer, HER2-targeted drug delivery

## Abstract

**Layman summary:**

HER2 is an oncogenic driver in a subset of breast cancer. Despite the fact that there are the options of several anti-HER2 targeted therapies, most patients with metastatic HER2+ breast cancer die from the disease. Therapies to overcome treatment resistance in the metastatic settings (including brain metastasis) are actively being pursued. Recently, cell cycle inhibitors (CDK 4/6 inhibitors) have been approved to manage hormone receptor-positive breast cancer, and have encountered tremendous success. The cell cycle signaling proteins, Cyclin D-CDK4/6, are downstream of HER2 and play a key role in cellular proliferation. Moreover, cell cycle inhibitors have the capacity to cross the blood–brain barrier. Here, we review the published literature with regard to the rationale for CDK4/6-directed therapies in HER2+ breast cancer.

**Abstract:**

The development of HER2-targeted therapies has dramatically improved patient survival and patient management and increased the quality of life in the HER2+ breast cancer patient population. Due to the activation of compensatory pathways, patients eventually suffer from resistance to HER2-directed therapies and develop a more aggressive disease phenotype. One of these mechanisms is the crosstalk between ER and HER2 signaling, especially the CDK4/6-Cyclin D-Rb signaling axis that is commonly active and has received attention for its potential role in regulating tumor progression. CDK 4/6 inhibitors interfere with the binding of cell-cycle-dependent kinases (CDKs) with their cognate partner cyclins, and forestall the progression of the cell cycle by preventing Rb phosphorylation and E2F release that consequentially leads to cancer cell senescence. CDK 4/6 inhibitors, namely, palbociclib, ribociclib, and abemaciclib, in combination with anti-estrogen therapies, have shown impressive outcomes in hormonal receptor-positive (HR+) disease and have received approval for this disease context. As an extension of this concept, preclinical/clinical studies incorporating CDK 4/6 inhibitors with HER2-targeted drugs have been evaluated and have shown potency in limiting tumor progression, restoring therapeutic sensitivity, and may improving the management of the disease. Currently, several clinical trials are examining the synergistic effects of CDK 4/6 inhibitors with optimized HER2-directed therapies for the (ER+/-) HER2+ population in the metastatic setting. In this review, we aim to interrogate the burden of HER2+ disease in light of recent treatment progress in the field and examine the clinical benefit of CDK 4/6 inhibitors as a replacement for traditional chemotherapy to improve outcomes in HER2+ breast cancer.

## 1. Introduction

Breast cancer (BC) is one of the most frequently diagnosed cancers in women, with approximately 2.3 million new cases diagnosed worldwide in 2020 [1]. In the United States, 1 in every 8 women (13%) is at risk of developing BC in their lifetime, and 1 in every 39 women (3%) is at risk of dying from BC [2,3]. While BC may commonly be perceived as a single disease, it is heterogeneous and phenotypically diverse, with a prognosis widely dependent on the underlying genetic aberration(s) [4]. Based on the molecular subtypes (gene expression), BC can be classified into four main molecular subtypes: Luminal A (ER+ (ER: estrogen receptor] and/or PR+ (PR: progesterone receptor), HER2− (HER2: human epidermal growth factor receptor 2), and low Ki67 (<14%)); Luminal B (ER+ and/or PR+ and HER2+(luminal HER2 group); ER+ and/or PR+, HER2−, and high Ki67 (>14%)); HER2-enriched (ER−, PR−, and HER2+); and basal-like or triple-negative (TNBC) (ER−, PR−, HER2−, and CK5/6 and/or EGFR+) [5]. The distinct biological features of each of these subtypes can be exploited to develop effective treatment strategies. HER2+ BC is particularly an aggressive form of BC, accounting for nearly 15–20% of all BC cases, and is historically associated with poor outcomes and risk for disease relapse [6]. It typically overexpresses HER2 transmembrane receptor tyrosine kinase (RTK) through *ERBB2* gene amplification. 

The HER2 signaling network and its complex relationship with the downstream signaling, including the cell cycle pathway (CDK4/6-CylinD1-RB1 axis), explains the acquired resistance to anti-HER2 therapy frequently observed in clinics. The mitogenic signals mediated by HER2 and estrogen receptor (ER) converge to the Cyclin D1-CDK4/6 complex, the key regulator of the G1-S transition for cell cycle progression. The HER (*ERBB*) family of RTK (HER1/HER2/HER3/HER4) consists of a glycosylated extracellular ligand-binding ectodomain (leucine- and cysteine-rich subdomains), a single transmembrane *α*-helix, and an intracellular (cytosolic) region comprising a short juxtamembrane segment, tyrosine kinase domain, and a tyrosine-containing flexible C-terminal tail [7,8]. The extracellular receptor domain is known to be activated by ~11 peptide ligands and, upon stimulation, either homo- or hetero-dimerize to activate the downstream tyrosine kinase signal transduction pathways that result in cell proliferation, motility/adhesion, and resistance to apoptosis: all key features of cellular malignancy [9]. HER2 does not have a *bona fide* ligand, it can homo-dimerize (upon amplification/overexpression), and it forms a more stable complex with other HER receptors (hetero-dimerization). HER3 has impaired kinase activity; however, the dimerization of HER2 and HER3 activates its kinase domain resulting in one of the worst known BC prognoses [10]. HER3 contains six consensus binding sites for the YXXM motif and, therefore, has the highest mitogenic potential to activate the PI3K/AKT pathway through the binding of p85—the regulatory subunit of PI3K [11]. The amplification of *ERBB2* serves as a predictive and prognostic marker and is typically associated with tumor aggressiveness (high-grade tumor mitotic count), resistance to chemotherapy, distant metastases, and adverse clinical outcomes [12]. HER2 is the only clinically validated marker to identify BC patients for anti-HER2 treatments; however, it is becoming increasingly evident that HER2+ BC is a heterogeneous disease. Amplification of HER2 increases ligand-independent receptor homodimerization, as well as ligand-dependent heterodimerization (HER2-HER3) in the HER2-amplified tumors. A heterodimer containing HER2 and HER3 relies on HER3 for signaling. The importance of HER3 may be, in part, related to its potent ability to activate the downstream PI3K-AKT-mTOR pathway [13,14,15]. In contrast, the HER2 homodimer lacks phosphorylated tyrosine in its cytoplasmic tail, preventing it from docking to the PI3K pathway adaptor protein, p85, and activating the PI3K signaling. The engagement of the HER2 homodimer primarily activates the GRB2 and SHC adaptor proteins that initiate RAS/MAPK signaling [16], which is also known to activate p110α (the catalytic subunit of PI3K) [17]. It has also been known that GAB1, an adaptor protein, plays a role in HER3 independent activation of the PI3K pathway. Homodimerization/auto-phosphorylation of HER2 binds with GRB2 (a *bona fide* adaptor protein for the activation of the RAS-MAPK pathway), which has the capacity to bind with another adaptor protein called GAB, and leads to the downstream activation of PI3K-AKT-mTOR pathway following recruitment by GRB2 [18,19,20,21]. Furthermore, EGFR and HER2 heterodimerization allow EGFR to be recycled rather than degraded (typically occurs on activation), promoting sustained signaling capabilities [19,22]. HER2 signaling pathway potentially creates an oncogenic addiction and renders sensitivity to HER2 inhibition, along with its downstream effectors to targeted agents or chemotherapy (e.g., paclitaxel/docetaxel).

The introduction of HER2-directed therapies (monoclonal antibody/antibody-drug conjugates/tyrosine kinase inhibitors) has dramatically shifted the treatment landscape of HER2+ BC, and changed the natural history of the disease progression [23]. The key receptor status (HR [hormonal receptor] and HER2 positivity) is widely probed in BC and currently guides the selection of a particular targeted therapy. HER2+ BC remains one of the most investigated diseases, resulting in the development of several targeted agents in the last decade. A list of currently approved therapies for the treatment of HER2+ BC has been provided (Table 1). There have been no reports so far of *ESR1* mutation in *ERBB2* (*HER2*)-amplified BC. *ESR1* mutations have been identified in HR+ BC, predominantly in metastatic cases (~30%) that cause estrogen-independent functioning of ER, and are linked to the development of therapeutic resistance [24]. The primary treatment paradigm at presentation (depending on the disease stage) for HER2+ BC includes surgical resection, preceded or followed by a course of targeted therapies and combinatorial chemotherapies—though an effort is being made to reduce reliance on chemotherapy. It may also be combined with other therapies that target imbalanced oncogenes and tumor suppressors of MAPK/PI3K pathways, such as *PIK3CA*, *PTEN*, and *AKT*, which are frequently co-altered (e.g., mutated) in BC. Conventional chemotherapy imposes a heavy toll of toxicities on BC patients, and the continued casualties from HER2+ BC require new treatment alternatives to be explored. Resistance to targeted therapies remains a major concern in a subset of this population. In particular, ER signaling and its crosstalk with the HER2 signaling pathway is one of the most common and clinically proven resistance mechanisms in ER+/HER2+ BC [25,26]. Recently, cell cycle inhibitors, viz., palbociclib, ribociclib, and abemaciclib, have been successful in targeting ER+ disease and have gained FDA approval for use as a monotherapy or in conjunction with other endocrine therapies for advanced or metastatic BC [27]. Importantly, the PI3K-AKT pathway is frequently dysregulated in HER2+ BC and affects gene transcription (*CCND*/*CCNE*), and deactivates regulatory proteins (p27^Kip1^/p21^Cip1^) of cell cycle machinery that facilitates uncontrolled cell proliferation and oncogenic transformation [28]. Similarly, phosphorylated ERK1/2 (a downstream molecule of the MAPK pathway) translocates to the nucleus and activates transcriptional factors of multiple genes, such as *c-Myc*, *Elk-1*, *STAT*s, *Jun*, *Fos*, etc., which are essential for cell growth, proliferation, and differentiation [29]. The PI3K and MAPK pathways cooperate and cross-regulate various aspects of gene transcription and post-translational modifications that promote cell cycle progression (via upregulation of *CCND1* and related genes), adhesion/motility, and resistance to apoptosis. Preclinical evidence suggests synergy between CDK 4/6 inhibitors (CDKi) and anti-HER2 therapies, including their efficacy in trastuzumab-resistant settings [30,31]. Hence, targeting cell cycle progression with CDKi might be an effective option along with HER2-targeted therapy for the treatment of *ERBB2*-amplified BC. The CDKi competitively binds to the ATP binding pocket of CDK 4/6 and prevents its association with CCND (1, 2, and 3), thereby, preserving Rb protein in its native (active) conformation (unphosphorylated state) that leads to G1 phase arrest, interference with cell cycle progression, and subsequent senescence/apoptosis [32]. The action of CDKi in compensating the defects of the cell cycle regulatory mechanism and restraining cell division has provided a strong rationale to evaluate the therapeutic benefits of CDKi in the context of HER2+ BC too. 

The purpose of this review is to provide brief insights into HER2-directed therapies with a focus on CDKi for HER2+ BC and to evaluate its significance for chemotherapy-free treatment strategies. Brain metastases represent a substantial clinical challenge for patients with HER2+ BC, with an incidence of up to 30—50% [33,34]. Recent data showed that tucatinib, along with trastuzumab, can cross the blood–brain barrier and has significantly improved the progression-free survival (PFS) and overall survival (OS) in the HER2+ brain metastatic subset [35]. Similarly, preclinical models and emerging clinical evidence suggests that cell cycle inhibitors can also penetrate the blood–brain barrier and target central nervous system metastases in Rb-proficient tumors [36,37,38,39,40]. Tolaney et al. reported an intracranial objective response rate of 24% associated with abemacicilib administration in a phase II study of patients with brain metastases secondary to HR+/HER2-metastatic BC (MBC) [40]. Likewise, Tien and co-workers showed good CNS penetration with ribociclib in a phase 0 clinical trial of glioblastoma patients (*N* = 12) with *CDKN2A* deletion or *CDK4/6* amplification [39]. With this in mind, we argue that there is a good opportunity to improve outcomes in the HER2+ brain metastatic subset following treatment with trastuzumab, pertuzumab, and a cell cycle inhibitor, especially in ER+/HER2+ BC patients. Our review article advocates the concomitant blockade of HER2, CylinD-CDK4/6-RB pathway, and/or ER as an effective chemotherapy-free treatment opportunity for HER2+ BC patients.

## 2. Cell Cycle and Cell Cycle Inhibitors

The cell cycle is a series of events that leads to the duplication and division of cells. It integrates a continuous phase of cell growth (increase in cytoplasmic mass) with a discontinuous phase of DNA synthesis and division. The cell cycle is a tightly regulated event and an orderly progression through the phases of growth (G), synthesis (S), and differentiation (M/C, mitosis, and cytokinesis) that results in the formation of two identical daughter cells. The Cyclins–CDK complexes play a pivotal role in coordinating the transition through various phases of the cell cycle. It has been shown that in various cancers, including the breast, the genes *CCND*/*CDK*s are frequently mutated, which allows spontaneous association between CCND/CDKs [56]. Cyclin D-CDK4/6 complex formation is a key event for the initiation of the cell cycle and is essential for transitioning through the G1 restriction point into the S phase. Hence, a dysregulated or more frequent association between Cyclin D-CDK predisposes cell cycle machinery to undergo uncontrolled proliferation leading to tumorigenesis. The estrogen receptor performs a dual job: as a receptor in the nucleus (for growth and proliferation in response to hormones; estrogen upon reception) and as a transcription factor, which can directly translocate into the nucleus and promote upregulation of *CCND* genes [57,58]. 

**Figure 1:**
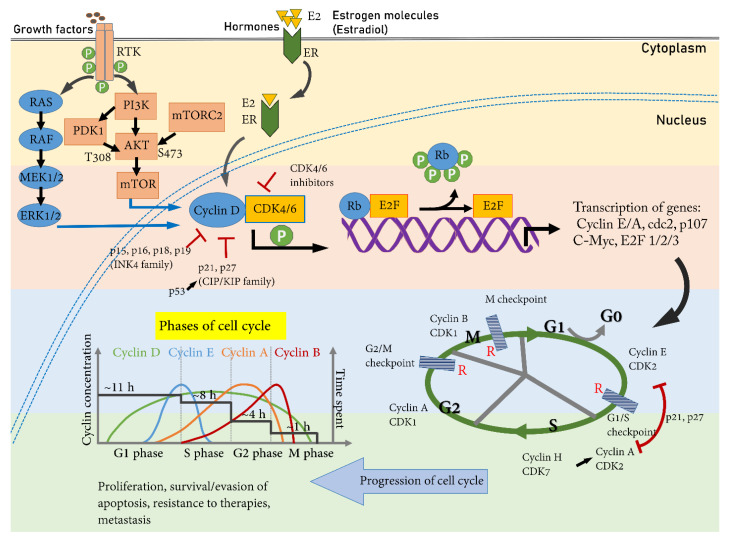
Cell cycle progression via upregulation of Cyclin D and Rb phosphorylation. Cyclin D is a downstream target of the estrogen receptor. Upon estrogen (estradiol β, E2) reception, ER translocates from cell membrane to nucleus and increases the expression of cyclin D through activation of target genes. Cyclin D is also upregulated by MAPK (via ERK1/2) and PI3K (via mTOR) signaling upon growth factor stimuli. MAPK and PI3K cooperate to increase the transcription of Cyclin D. The binding of Cyclin D with its cognate partner CDK 4/6 phosphorylate Rb causes the release of E2F (E2F activation—a prerequisite step) and activates cell cycle responsive genes for progressing into the S phase. The entry into the successive phase of the cell cycle is guarded via restriction points, which check the appropriateness and readiness of the cell for cell cycle progression and division. The level of cyclins “oscillate” at different phases of the cell cycle and binds with their CDK partner in a time-dependent manner. The duration of each phase of the cell cycle varies, with the maximum time spent in the G1 phase and the minimum time spent in the M phase (shown for a cycle time of 24 h [61]).

Once the cell passes through the G1/S restriction point, it is more likely committed to cell division. The G1/S restriction point is controlled by an Rb tumor suppressor protein (encoding gene: *Rb*), which in an unphosphorylated state binds tightly to the transcription factors–E2F (E2F- 1, 2, 3) and prevents progression into the S phase [59]. To overcome this restriction, Cyclin D and CDK 4/6 form a complex and phosphorylate Rb, causing a partial release of E2F. E2F then causes activation of genes necessary for transcription of Cyclin E. Cyclin E with its partner CDK2 further phosphorylates Rb (Rb hyper-phosphorylation), and this results in the complete release of E2F from the Rb [60]. Hence, the inhibition of the Cyclin D-CDK4/6-Rb-E2F pathway provides a viable mechanism to plug rapid cell proliferation and holds a substantial promise for cancer treatment. A brief schematic of signaling cascades that lead to the initiation and progression of the cell cycle has been depicted (Figure 1).

## 3. Approved CDKi

Cell cycle inhibitors have been approved in the context of ER+ metastatic BC (as first-line therapy). These cancers were previously treated with systemic chemotherapy and endocrine therapy, which affects the functioning of hormonal receptors. However, resistance to hormonal therapies and disease progression is a common occurrence, and these tumors often harbor amplification of *CCND1* and overexpression/mutation of *CDK4/6*. It has previously been reported that cell cycle pathway genes are upregulated (*CCND1*–38%, *CDK4*–24%) in HER2-enriched BC [62]. Preclinical studies in murine models have shown that both *CCND1* and *CDK4* are necessary for tumorigenesis of HER2+ BC [63,64]. Recently, Goel and colleagues showed that Cyclin D1-CDK4 mediates resistance to targeted therapy in HER2+ BC using transgenic mouse models, cell-line-based mechanistic studies, and clinical specimens. Most importantly, the resistance was reversed upon treatment with CDKi and HER2-targeted therapy [31]. Currently, three CDKi have been approved by the US-FDA for use in ER+ disease based on the impressive results of preclinical and clinical studies. These drugs have shown good potency and manageable toxicity, and are routinely prescribed in conjunction with hormonal therapies [65,66,67]. 

## 4. Palbociclib

Palbociclib is the first CDK 4/6 targeted drug to be approved by the FDA for use in metastatic ER+ HER2- disease in conjunction with hormonal therapy. It is taken orally and has an enzymatic IC50 of approximately 11 nM for CDK4 and 15 nM for CDK6 [68]. In 2015, palbociclib was granted accelerated approval by the FDA to treat ER+/HER2- MBC in combination with letrozole as initial endocrine-based therapy in postmenopausal women. The indication was expanded in 2016; palbociclib with fulvestrant was approved for use in HR+/HER− advanced or metastatic BC with a disease progression following endocrine therapy. Following the results of the phase 3 PALOMA-2 trial, the FDA, in 2017, granted regular approval to palbociclib and expanded its use as first-line therapy in combination with an aromatase inhibitor in postmenopausal women with HR+/HER2− advanced or metastatic BC. Recently, in 2019, the FDA approved palbociclib in combination with an aromatase inhibitor as the first-line treatment for men with HR+ and HER2− metastatic BC. Neutropenia, leukopenia, fatigue, and anemia are the most common adverse events associated with palbociclib therapy [69].

## 5. Ribociclib

Ribociclib is the second CDK 4/6 targeting a small molecule inhibitor. It is administered via the oral route and has an enzymatic IC50 of approximately 10 nM for CDK4 and 39 nM for CDK6 [68]. It has recently been reported that the free drug availability of ribociclib is higher compared to palbociclib and abemaciclib, which makes it rapidly available in the blood circulation for cellular uptake and CDK4 inhibition [70]. In cellular assays, ribociclib has shown an 8-fold higher efficacy on CDK4 than on CDK6 [70,71]. It has also been noted that BC has amplified *CDK4* levels, while lymphoid tumors have high CDK6 levels [72]. The FDA approved ribociclib in 2017, based on the MONALEESA-2 trial, in combination with an aromatase inhibitor, as an initial endocrine-based therapy for the treatment of postmenopausal women with HR+/HER2− advanced or metastatic BC. In 2018, ribociclib received additional indications for combination with an aromatase inhibitor for the treatment of pre-, peri- or postmenopausal women as initial endocrine-based therapy. It is also indicated for postmenopausal women in combination with fulvestrant as an initial endocrine-based therapy (first-line) or following disease progression on endocrine therapy (second-line). Similar to palbociclib’s mechanism of action, ribociclib induces tumor regression and/or apoptotic cell death in a dose-escalated manner following treatment in neoplasms [73]. Neutropenia and leucopenia are the most frequent grade 3 or 4 adverse events reported with ribociclib therapy [66]. 

## 6. Amebaciclib

Abemaciclib is the most recently approved drug in this category and is also taken orally. It is highly potent among all other approved CDKi, as evidenced by its lowest half-maximum inhibitory concentration (IC50) of 2 nM and 10 nM for CDK 4 and 6, respectively [36,68]. Evidence suggests amebaciclib can penetrate the blood–brain barrier and, therefore, can confer significant therapeutic benefit to ER+/HER2− BC patients diagnosed with brain metastases [40]. It is currently administered orally, twice a day. The FDA approved abemaciclib in 2017 and indicated for combination with fulvestrant in HR+/HER2− MBC with disease progression, following endocrine therapy and as a monotherapy with disease progression following endocrine therapy, and prior to chemotherapy in the metastatic setting. In 2018, the FDA expanded its approval for combination with an aromatase inhibitor as initial endocrine-based therapy for the treatment of postmenopausal women in HR+/HER2- advanced or metastatic BC. The approval follows the safety and efficacy of the MONARCH-3 trial. The most frequent grade ≥ 3 adverse events that occurred with abemaciclib therapy are neutropenia, diarrhea, and leukopenia [74].

## 7. CDKi in HER2+/ER+ BC

Because of the existence of common and redundant growth factor signaling networks, the interest has expanded to test the efficacy of CDKi in the ER+/HER2+ domain too. Resistance to ER or HER2-targeted therapies remains a major concern in tumor-relapse/refractory cases, and effort is being made to understand the mechanism of resistance and to re-sensitize or obstruct disease progression through the use of novel therapies and/or combinatorial treatment approaches [30,75,76]. The defects in tumor suppressors, oncogenes, and host genetics inspire the transformation of a normal cell into a neoplastic cell. It results in repeated cell division, invasion, and destruction of native tissues, and loss of tissues’ physiological function (malignancy). CDK 4/6 with partner cyclins (Cyclin D1/D2/D3) play a key role in orchestrating cell proliferation; they coordinate transitioning through initial checkpoints of the cellcycle phase. CDKi have been approved for use in HR+ MBC based on the impressive PFS data and low toxicity from key trials [65,66,69,74]. The ER and HER2 pathways, independently or collectively, converge to upregulate genes and bypass regulatory brake(s) for facilitating cell proliferation. Hence, CDKi, if used in conjunction with endocrine and HER2-directed therapies, may potentially provide synergistic pharmacological benefits. Cyclin D1 is a downstream target of HER2 signaling, especially in the ER+/HER2+ context [77,78]. Preclinical studies have shown the efficacy of CDKi in targeting ER+/HER2+ or ER−/HER2+ BC along with HER2-targeted therapy [30,79]. 

Currently, several clinical trials are underway that examine synergistic pharmacological use of CDKi in HER2+ BC (Table 2) with other signaling molecule inhibitors. Data from these trials may reveal a new strategy for the treatment and diagnosis of ER+/HER2+ BC. 

## 8. CDKi in HER2+/ER- BC

**Figure 2:**
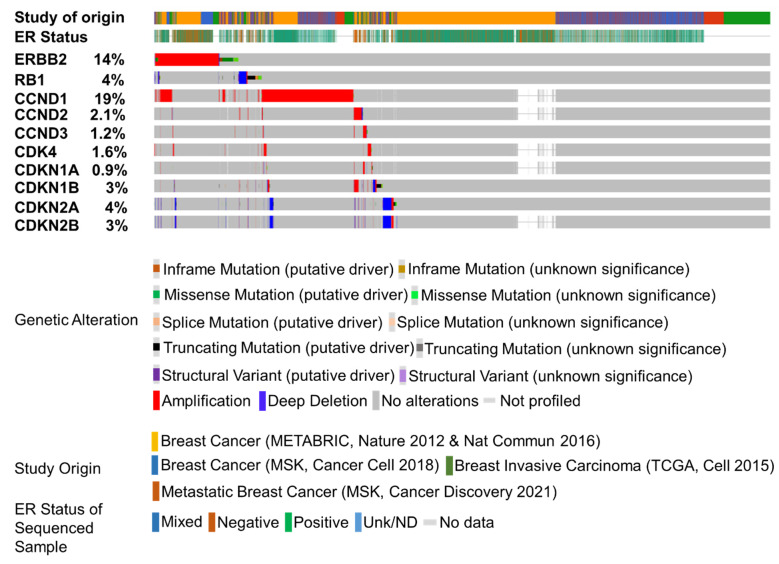
Somatic alterations associated with cell-cycle-related genes in breast cancer genomic datasets. Oncoprint representing the status of 10 genes—*ERBB2*, *RB1, CCND1*, *CCND2*, *CCND3, CDK4, CDKN1A*, *CDKN1B*, *CDKN2A*, and *CDKN2B*—in 6198 patients/6609 samples in four studies: Breast Cancer (METABRIC, Nature 2021 & Nat Commun 2016) [94,95,97], Breast Cancer (MSK, Cancer Cell 2018) [96], Breast Invasive Carcinoma (TCGA, Cell 2015) [93], and Metastatic Breast Cancer (MSK, Cancer Discovery 2021). Queried genes are altered in 2444 (39%) of queried patients and 2600 (39%) of queried samples. Genetic alterations are depicted. We acknowledge cBioPortal for Cancer Genomics (https://www.cbioportal.org/, accessed on 30 November 2021), which provides a web resource for exploring, visualizing, and analyzing multi-dimensional cancer genomics data [100,101]. We acknowledge TCGA Research Network for generating the TCGA datasets (https://www.cancer.gov/tcga, accessed on 30 November 2021).

There has been an incredible success in targeting ER+ disease with CDKi [86]. The genes regulating the cell cycle, *CDK 4/6* and cyclins, are redundantly expressed in multiple cancers, and their expression levels closely correlate to patient outcomes with moderate to high gene amplification, often leading to poor prognosis [87]. The potential advantage of CDKi may also lie when combined with other targeted therapies, particularly in HER2+ BC, for overcoming treatment resistance [31]. Besides ER-mediated Cyclin D expression, its upregulation and deactivation of major cell cycle regulatory proteins are largely facilitated through other classical tumor signaling pathways- MAPK and PI3K pathways [88]. These pathways are often upregulated and remain in a “switched-on” position in HER2+ BC that serve to promote aggressive malignancy. The MAPK pathway activates Myc and Jun/Fos transcription factors that increase the synthesis of Cyclin D [89]. Similarly, the PI3K–AKT pathway through AKT phosphorylation inactivates p27, an onco-suppressor protein responsible for arresting the entry to the G1/S phase of the cell cycle [90]. The p27 inactivation results in activation of CCNE1-CDK2 via CCND1/CDK4/6, which facilitates transitioning of the cell cycle through the restriction points [91]. Downstream of AKT, mTOR can activate the translocation of CCND1 mRNA, resulting in the formation of CCND1/CDK4 and CDK6 complexes (CCND1 mRNA translation via mTOR) [92] (Figure 1). Recent large-scale cancer genomic datasets show the overlap between *CCND1* amplification and *ERBB2* amplification in breast cancer patients (Figure 2) [93,94,95,96,97]. Additionally, in *ERBB2* amplified tumors, *CCND1* amplification rarely overlaps with the mutations in *RB1* and/or CDK-inhibitors, *CDKN1A*, *CDKN1B*, *CDKN2A*, and *CDKN2B*, which predict a heightened sensitivity of CDKi in these tumors. Therefore, the use of CDKi in HER2+ BC seems to have a prima facie potency to check cell proliferation and obstruct neoplastic transformation. In addition, cell cycle inhibitors have recently received attention/clinical focus across multiple cancer disciplines and hold the promise of becoming a “broad-spectrum” anticancer therapy for various combinatorial strategies [87,98,99].

## 9. Towards a Common Goal of a Chemotherapy-Sparing Treatment Plan

Because of the heavy side effects from the administration of systemic chemotherapy, recent studies have begun to focus on adopting a chemotherapy-free, de-escalated treatment program using a combination of cell cycle inhibitors and targeted therapies (newly developed or existing). Historically, ER+ diseases are less aggressive than HER2+ or triple-negative counterparts and more sensitive to cell cycle inhibition, providing an opportunity to treat this category of BC (ER+/HER2+) exclusively with hormonal and cell cycle inhibitors in conjunction with anti-HER2 therapy [59]. In addition, preclinical studies have shown that ER+/HER2+ diseases are also responsive to cell cycle inhibitors [30,31]. As a result, there has been a growing interest in treating ER+/HER2+ disease with a combination of cell cycle inhibitors and HER2-targeted therapies.

Several preclinical/clinical investigations have provided the clue that supports the expanded use of CDKi in HER2+ BC. The data to support CDK4/6 inhibitory action in *ERBB2* (HER2)-amplified breast tumors comes from the early work of Finn and colleagues [30], wherein in a panel of BC cell lines evaluated in vitro, PD 0332991 (palbociclib) was found to be most effective in inhibiting cell proliferation in luminal estrogen receptor-positive (ER+) subtypes, including *HER2*-amplified but ineffective in non-luminal/basal subtypes. The authors reported that treatment with PD 0332991 in those sensitive (luminal ER+/HER2-amplified) cell lines resulted in cell cycle arrest at the G0/G1 phase and blockade of Rb phosphorylation—a crucial event that thwarts upregulation of cell cycle genes by preventing *E2F* release. Genetic profiling studies of HER2+ tumors further revealed that CDK4/6i-sensitive cell lines had elevated levels of pRb and Cyclin D and reduced expression of *CDKN2A* (p16, a physiological inhibitor of CDK4/6–CCND1 complex), providing preliminary evidence that CDK4/6i may have a clinical opportunity in *HER2*-amplified BC. 

The Cyclin D–CDK4/6 pathway lies downstream of HER2-signaling, and its hyperactivation has been implicated in the development of acquired resistance to HER2-targeted therapies [31,102]. CDKi has shown a complementary mode of action with various HER2-inhibitors (Neratinib, Afatinib, and BMS-599626) in a dose-dependent fashion, potentially providing an avenue to treat refractory tumors using a combinatorial therapeutic approach. The additive action of PD-0332991 with these targeted therapies has been verified in different cell culture models of HER2+ BC [102]. 

Zhao and colleagues have shown synergistic effects of neratinib in HER2-overexpressing BC lines in vitro when combined with CDK4/6i, mTOR, and MEK inhibitors [103]. The combinatorial strategy was tested in HER2+ patient-derived xenografts (PDX) in mice models and found to have enhanced antitumor efficacy demonstrated by a decrease in tumor volume (tumor viability/growth) and extended event-free survival [103]. 

Abemaciclib has been shown to have anti-proliferative activity in ER+/HER2+ cell line-based models [104]. It was found to be particularly effective among cell lines that express high ER levels, unaltered Rb, and high total Rb, among other cellular factors. Its efficacy as a single-agent therapy has been confirmed in the xenograft model of HER2+/ER+ BC. When co-administered with an anti-HER2 agent (trastuzumab) and endocrine therapy (tamoxifen), it further elicited an enhanced response to tumor inhibition and regression that supports the use of CDK4/6i in combinatorial treatment strategies for ER+/HER2+ BC.

Similarly, palbociclib has shown a potent cytostatic effect on surgically resected specimens of BC in an ex vivo culture irrespective of ER or HER2 status. The pharmacological response could not be achieved in tissues that lacked Rb-expression or had high expression of p16^ink4a^ (a tumor suppressor protein regulating cell cycle) [75]. Furthermore, 13albociclib has shown a dose-dependent growth inhibition in preclinical models of HER2+ BC cell lines. The response was very high in cell lines that were sensitive to trastuzumab/pertuzumab (TP), such that the difference between albociclib + TP or single-agent 13albociclib therapy was indiscernible. In addition, albociclib maintained a dose-dependent synergy with TP in resistant cell lines [76].

### Clinical Trials Evaluating the Efficacy of Cell Cycle Inhibition in Combination with HER2-Directed Therapies

The randomized, three-arm, open-label, phase II **monarcHER trial** (*N* = 325, (intent-to-treat (ITT) population = 237), NCT02675231) examined the safety and efficacy of abemaciclib plus trastuzumab, with or without fulvestrant to standard-of-care single-agent chemotherapy (physician’s choice), plus trastuzumab in women with HR+/HER2+ locally advanced or metastatic BC [105]. Eligible patients were adult women, were pre-/perimenopausal (natural/surgical or ovarian suppressed, age ≥ 18 y), and were those who received taxane and T-DM1 in any disease setting and at least two HER2-directed therapies for the advanced disease. The study met its primary endpoint. The pre-specified primary analysis performed at 169 events demonstrated significantly longer PFS with the triplet combination (abemaciclib + trastuzumab + fulvestrant, Arm A) over the control arm (chemotherapy + trastuzumab, Arm C) (8.3 vs. 5.7 mo; hazard ratio (HR): 0.67). The study investigators found no significant difference in PFS between abemaciclib and trastuzumab treatment (Arm B) versus the control arm (5.7 vs. 5.7 mo., HR: 0.943). ORR in the ITT population was reported at 33%, 14%, and 14% in Arms A, B, and C, respectively. No new safety signals were found. The most common grade 3/4 adverse events across all arms were neutropenia (27%, 22%, and 26%; Arms A, B, C), leukopenia (10%, 3%, and 10%, respectively), thrombocytopenia (10%, 7%, and 3%, respectively), and diarrhea (9.0%, 7%, and 3%, respectively) [105].

The phase II **NA-PHER2 trial** (*N* = 36; 35 evaluable for safety, 30 for trial outcomes; *NCT02530424*) assessed the neoadjuvant chemotherapy-sparing treatment strategy for patients who express ER+/HER2+ BC by concurrently targeting ER, HER2, and RB1 signaling with the combination of trastuzumab and pertuzumab (anti-HER2), palbociclib, and fulvestrant [106]. The co-primary endpoints of the trial were longitudinal measures of Ki-67 and apoptosis at the baseline (before therapy), two weeks of treatment (Ki-67 only), and at the time of surgery. The secondary measures were pathological complete response (pCR), clinical objective response (as per mRECIST criteria), and safety/tolerability of the drug association. The study reported substantial potency of the drug combination for impeding tumor growth measured in terms of decreased Ki-67 expression (mean ± SD) from baseline (31.9 ± 15.7, *n* = 30) to two weeks of treatment (4.3 ± 15, *n* = 25, *p* < 0.0001), and at the time of surgery (12.1 ± 20, *n* = 22; *p* = 0.013). Prior to the surgery, 29 (97%; 95% CI 83-100) of 30 patients had a clinical objective response, and a pCR was observed in 8 patients (27%) in breast and axillary nodes. The most frequent adverse events (grade 3) included neutropenia (29%), diarrhea (14%), and stomatitis, increased alanine aminotransferase, and hypersensitivity reactions (3% each). The study reported no deaths or grade 4 or serious adverse events with the drug association [106]. While the study is an exploratory analysis with low participants, the results, including safety analysis, are highly encouraging with a potential for paradigm-shifting clinical care, and need to be further validated in a large-scale, randomized phase III clinical trial. 

The randomized, phase II **SOLTI-1303 PATRICIA trial** (*N* = 71, NCT02448420) has evaluated the safety and efficacy of palbociclib in combination with trastuzumab with/without endocrine therapy for the treatment of patients with locally advanced or metastatic HER2+ BC who have previously received 2–4 lines of anti-HER2-containing regimens for their advanced disease [107]. Eligible patients were adult females (age ≥ 18 y) with laboratory-confirmed invasive HER2-positive BC. Patients were randomized into ER- and ER+ cohorts: those who were ER− (cohort A) received treatment with trastuzumab and palbociclib; the ER+ population was further stratified into cohorts B1 or B2, and received treatment without or with letrozole, respectively. The study reported PFS at 6 months, the primary endpoint of the trial, to be 33.3% in cohort A, 42.8% in cohort B1, and 46.4% in cohort B2. Safety analysis showed that 97.7% of patients had grade 1–2 toxicity versus 84.4% with grade 3–4 toxicity that mostly included neutropenia (66.4%) and thrombocytopenia (11.3%). The PAM50 intrinsic tumor subtyping showed that the investigational treatment had a substantial beneficial effect on the luminal disease over the non-luminal subtype, demonstrated in terms of PFS (median PFS- 10.6 (luminal A or B) vs. 4.2 months (non-luminal); adjusted HR: 0.40; *p* = 0.003). The tolerable safety profile and relatively effective survival data for the PAM50 luminal A/B tumor subtype upfront identifies patients who may or may not be a candidate for this treatment strategy [107].

The safety and efficacy of palbociclib in combination with trastuzumab and letrozole for neoadjuvant treatment of ER+/HER2+ stage II-III BC has been evaluated in phase II of the **PALTAN trial** (*N* = 26) (*NCT02907918*) [108]. The study aimed to evaluate the efficacy of de-escalation of trastuzumab-containing regimens in the neoadjuvant setting (chemotherapy-sparing). The investigators sought to improve the therapeutic efficacy by modulating ER signaling with endocrine therapy, and treatment with CDKi plus trastuzumab. The primary objective was to show improvement of pCR with this combination. The secondary endpoint was the measure of safety and tolerability of the drug association. The trial was terminated due to futility, and the results were recently presented at the 2022 San Antonio Breast Cancer Symposium (SABCS) [108]. The trial drug combination resulted in a pCR of 7.7% and was well-tolerated. The most common treatment-emergent adverse events were leukopenia (7.7%), neutropenia (7.1%), and anemia (5.9%). RNA sequencing and Ki67 data from biopsy specimens collected at baseline, day 15, and surgery showed strong anti-proliferative effects of the triple-drug combination. Further results of this trial are awaited.

The safety and efficacy of palbociclib in combination with anti-HER2 plus endocrine therapy over anti-HER2 plus endocrine therapy (standard therapy) are being examined in the randomized, open-label, phase III **PATINA trial** (*N* = 496 (anticipated)) (*NCT02947685*) for patients (men/women) with HR+/HER2+ metastatic BC [82,109]). Currently, trastuzumab with pertuzumab and taxane is offered as a first-line therapy for the treatment of MBC. As a maintenance dosing, patients continue treatment with HER2-directed agents and endocrine therapy after completion of the chemotherapy regimen. The study hypothesized that the addition of a cell cycle inhibitor in the maintenance setting (after induction therapy) would enhance the synergistic benefits of standard HER2-targeted and anti-ER therapies for delaying the onset of therapeutic resistance and improving survival. Patients, after receiving 4–8 cycles of chemotherapy (taxane or vinorelbine) with anti-HER2 therapy, are randomized to receive anti-HER2 (trastuzumab +/− pertuzumab) with endocrine therapy, with or without palbociclib. The primary objective is to demonstrate the improvement of PFS with the addition of palbociclib in the standard maintenance therapy (anti-HER2 + anti-ER) after induction treatment for HR+/HER2+ MBC. The secondary endpoints include OS, ORR, DoR, clinical benefit rate, and safety. The trial is being conducted in major oncology research centers in Australia, New Zealand, the United States, Spain, and Germany. Oncologists are eagerly awaiting the results of the trial. 

Most recently, the data of a phase 1b trial in a very small cohort (*N* = 12) show promising clinical activity of ribociclib in combination with T-DM1 in HER2+ MBC patients regardless of their HR status (66.7% of patients had HR+/HER2+) [110]. While CDKi can impair the efficacy of T-DM1 by preventing cells from entering into the S or M phase [111], it can potentially be avoided with a non-overlapping dosing strategy [110]. This scheme can also mitigate toxicities common with both agents, e.g., thrombocytopenia. DM1 has a half-life of approximately 4 days [112]. T-DM1 was administered intravenously on day 1 of each 21-day cycle, followed by ribociclib on days 8–21 of a 21-day cycle. After a median follow-up of 12.4 months, the median PFS was 10.4 months, and the combination was generally well-tolerated, with the majority of toxicities reported in grades 1 and 2 [110]. 

Despite the development of targeted therapies against ER and HER2 receptors, tumor growth inhibition and subsequent eradication (complete response) has become a major therapeutic challenge. The intimate crosstalk between ER and HER2 signaling is fundamentally responsible for engendering escape strategies that provide resistance to therapeutic intervention. The continued use of an anti-HER2 backbone therapy is a common treatment approach in HER2+ MBC, but the potential long-term benefits from CDKi, especially in HER2+/ER− MBC, need to be explored further.

## 10. Future Directions

The innovations in HER2-targeted therapies have provided prolonged survival, offered hope, and significantly improved the natural course of the disease progression. The development of newer technologies (especially advances in next-generation sequencing) and the biological understanding of BC have provided insights into tumorigenesis and led to the discovery of various drug targets and their cooperative pathways. Despite these advancements, BC remains a major cause of death and suffering in adult females worldwide. HER2+ BC is one of the most aggressive forms of cancer, characterized by drug resistance, high-grade metastasis, and poor survival. At present, various anti-HER2 targeting agents have been approved for clinical use, while the efficacy of other novel agents and/or drug combinations are being tested in various clinical trials to search for a complete response. In a clinical setting (based on disease status), combinatorial targeted therapies with/without systemic chemotherapy are prescribed to patients, and this has dramatically improved prognosis. However, the development of early resistance to HER2-directed therapies remains a major treatment challenge. Despite witnessing several breakthrough discoveries in the field, patients progress to a point where no drugs are available for treatment, or drug combinations are equally ineffective. To date, MBC is an incurable disease and difficult to treat, and the quality of life is poorest in these patients. In addition, trastuzumab is not very effective for patients with brain metastases. Neratinib (a pan-HER inhibitor) has been associated with improved PFS and CNS outcomes in the HER2+ metastatic subset in the phase III NALA trial and was recently approved for the treatment of HER2+ MBC in combination with capecitabine [55]. Of note, biomarker analysis of the NALA trial showed that *PIK3CA* mutation was associated with shorter PFS, whereas higher HER2 expression (including patients with *HER2* mutation) was associated with longer PFS [113]. Previously, based on the results of the phase III ExteNET trial, single-agent neratinib has been approved for the extended adjuvant treatment of early-stage HER2+ BC after trastuzumab-based adjuvant therapy [54]. Since CDKi also has the capacity to cross the blood–brain barrier, we may argue that *de novo* brain metastatic *HER2*-amplified BC patients can be treated with trastuzumab, and pertuzumab plus CDKi.

According to the new ASCO guideline update for HER2+ BC patients, trastuzumab, pertuzumab, and a taxane are recommended for the first-line treatment, and trastuzumab deruxtecan for the second-line treatment. In the third-line setting, several treatment options exist, and clinicians should offer HER2-targeted therapies based on multiple factors. Current treatment options include regimens with tucatinib, trastuzumab emtansine (T-DM1), or trastuzumab deruxtecan (if not previously administered), and neratinib, lapatinib, chemotherapy, margetuximab, hormonal therapy, and a cell cycle inhibitor plus trastuzumab, plus fulvestrant [114]. Since there are no head-to-head trials comparing these regimens, it is difficult to recommend one therapy over the other. The patient and the clinician should discuss differences in treatment schedule, route of administration, co-morbidities, toxicities, emerging evidence, etc., during the decision-making process to identify the optimal treatment strategy. 

The remarkable clinical benefits of CDKi, along with other matched therapies and its relevance, have sparked interest beyond the realm of BC, including in the non-small cell lung, colorectal or pancreatic cancers (*KRAS*-mutant) [98,115,116], pediatric cancers [117], ovarian [118]/endometrial cancers [119], and other solid tumors that exhibit loss of cell cycle regulation due to downregulated/mutated tumor suppressor genes (*CDKN2A, CDKN2B*) and overexpression of many of the cell-cycle-associated oncoproteins such as Cyclin D, as well as cyclin-dependent kinases [120,121]. The applicability of CDKi across various neoplasms may potentially emerge as a “broad-spectrum” anticancer therapy and transform future clinical practice. The biomarkers and molecular/genetic profile common in these cancers are being studied, and it may help identify a subset of patients who may have the greatest therapeutic benefit with optimized treatment strategies, including treatment with cell cycle inhibitors. 

## 11. Conclusions

Published studies and data from multiple clinical trials support the concomitant blockade of HER2/ER and CDK4/6–Cyclin D–Rb pathways as an effective chemotherapy-free treatment opportunity for improved outcomes and management in HER2+/ER+ BC patients [30,31,106,122]. Furthermore, it has recently been shown by Tolaney and co-workers that abemaciclib plus fulvestrant is effective regardless of *PIK3CA* status, as *PIK3CA* is often mutated in HER2+ BC [123]. The success of treating HER2+ BC patients with cell cycle inhibition therapies encourages further exploration of rational treatment combinations with these inhibitors. It may likely provide a substantial clinical benefit by counteracting the resistance that is expected to evolve with the current therapies. The growing interest in cell cycle inhibitors and their proven efficacy in controlling breast tumors are predicted to revolutionize BC treatment and its prognosis, and provide sustained therapeutic benefits to HER2+ BC patients, especially those with aggressive and non-responding tumors. Recently, deep learning-based classification approaches have been used to predict novel drug targets associated with breast cancer pathogenesis (reviewed in [124]). We anticipate that in the near future, artificial intelligence (AI)/machine learning (MI) may leverage a greater role in predicting candidate *ERBB2*/HER2-amplified patients that can derive maximum clinical benefits from cell cycle inhibitor plus anti-HER2 therapies over traditional anti-HER2-based (chemotherapeutic) regimens.

## Figures and Tables

**Table 1:** FDA-approved molecularly targeted therapies in HER2+ BC.

Agent	Molecular Target	Key Trial	Indication (Approval Date)
Monoclonal antibody			
Trastuzumab	HER2 (Subdomain IV)	Phase II [41] and phase III Herceptin trials [42] NSABP B-31 (NCT00004067) NCCTG N9831 (NCT00005970) [43]	1st line: HER2+ MBC with paclitaxel (1998). 2nd/3rd line: Monotherapy for HER2+ MBC with ≥1 chemotherapy regimens for metastatic disease (1998). Adjuvant: In combination with doxorubicin, cyclophosphamide, and paclitaxel for the treatment of node-positive HER2+ BC (2006).
Pertuzumab	HER2 (Subdomain II)	Metastatic: CLEOPATRA (NCT00567190) [44] Neoadjuvant: NeoSphere (NCT00545688) [45] Adjuvant: APHINITY (NCT01358877) [46]	1st line: HER2+ BC (metastatic (2012) or neoadjuvant (2013)) with trastuzumab and docetaxel (triplet therapy). Adjuvant treatment of HER2+ early BC (EBC) in combination with trastuzumab and chemotherapy (2017).
Margetuximab	HER2 (with Fc-engineered region)	SOPHIA (NCT02492711) [47]	3rd line: Metastatic HER2+ BC with ≥2 anti-HER2 regimens with at least one for metastatic disease (2020).
Antibody-drug conjugate (ADC)			
Ado-trastuzumab emtansine (T-DM1)	HER2 (with microtubule inhibitor)	EMILIA (NCT00829166) [48] TH3RESA (NCT01419197) [49] KATHERINE (NCT01772472) [50]	2nd line: (Monotherapy) MBC was previously treated with trastuzumab and a taxane (2013). (Monotherapy) Adjuvant treatment for HER2+ EBC with the residual invasive disease after neoadjuvant taxane and trastuzumab-based treatment (2019).
Trastuzumab deruxtecan (DS-8201)	HER2 (with topoisomerase I inhibitor)	DESTINY-Breast01 (NCT03248492) [51] DESTINY-Breast03 (NCT03529110) [52]	3rd line: (Monotherapy) Metastatic HER2+ BC who have received two or more prior anti-HER2-based regimens in the metastatic setting (2019). 2nd line: (Monotherapy) Metastatic HER2+ BC previously treated with anti-HER2 antibodies and a taxane in the metastatic setting or (neo) adjuvant setting (2022).
Small molecule inhibitors (Tyrosine kinase inhibitors [TKI])			
Lapatinib	EGFR/HER1, HER2	Phase III Lapatinib trial (NCT00078572) [53]	2nd line: HER2+ MBC who have received prior therapy including an anthracycline, taxane, and trastuzumab (2007).
Neratinib	Pan-HER (EGFR/HER1, HER2, HER4)	ExteNET (NCT00878709) [54] NALA (NCT01808573) [55]	Extended adjuvant treatment of early-stage HER2+ BC (2017). 3rd line: Metastatic HER2+ BC who have received two or more prior anti-HER2-based regimens for their metastatic disease (2020).
Tucatinib	HER2	HER2CLIMB (NCT02614794) [35]	3rd line: Metastatic HER2-positive BC, including patients with brain metastases, with one or more prior anti-HER2 regimens in the metastatic setting (2020).

**Table 2:** Clinical trials with CDK4/6 inhibitors (CDKi) in HER2+ breast cancer **.

Trial (NCT ID)	Arms	Phase, Expected Enrolment & Site	Primary Outcome	Setting
CDKi, ribociclib in combination with trastuzumab Or T-DM1 for advanced/metastatic HER2-positive breast cancer (*NCT02657343*) [80]	Ribociclib+T-DM1 Ribociclib + Trastuzumab Ribociclib + Trastumzab + Fulvestrant	Phase Ib/II *N* = 25 USA	Maximum Tolerated Dose (MTD) and/or recommended Phase2 Dose (RP2D) Clinical Benefit Rate (CBR)	Metastatic
Ribociclib with trastuzumab plus letrozole in postmenopausal HR+, HER2-positive advanced breast cancer patients (*NCT03913234*)	Letrozole + Trastuzumab + Ribociclib	Phase Ib/II *N* = 95 South Korea	Progression-free survival	Metastatic
T-DM1 and Palbociclib for Metastatic HER2 Breast Cancer (*NCT03530696*)	T-DM1 + Palbociclib	Phase II *N* = 46 USA	Progression-free survival	Metastatic
Neoadjuvant treatment with palbociclib and exemestane plus trastuzumab and pyrotinib in ER-positive, HER2-positive breast cancer (neoPEHP) (*NCT04858516*)	Palbociclib + Exemestane + Trastuzumab + Pyrotinib	Phase II N=57 China	Pathological complete response	Neoadjuvant
To reduce the use of chemotherapy in postmenopausal patients with ER-positive and HER2-positive breast cancer (TOUCH) (*NCT03644186*) [81]	Control arm: Paclitaxel + Trastuzumab + Pertuzumab Palbociclib + Letrozole + Trastuzumab + Pertuzumab	Phase II *N* = 144 Belgium, France, Italy, Switzerland	Pathological complete response	Neoadjuvant
Palbociclib, trastuzumab, lapatinib, and fulvestrant treatment in patients with brain metastasis for ER-positive, HER2-positive breast cancer (*NCT04334330*)	Palbociclib + Trastuzumab + Lapatinib + Fulvestrant	Phase II *N* = 34 China	Objective response rate in the CNS	Metastatic (Brain)
Clinical Study of the Targeted Therapy, Palbociclib, to Treat Metastatic Breast Cancer (PATINA) (*NCT02947685*) [82]	Palbociclib + anti-HER2 therapy (Trastuzumab/Pertuzumab) + Endocrine Therapy (Letrozole, Anastrozole, Exemestane OR Fulvestrant) Control arm: Anti-HER2 therapy (Trastuzumab/Pertuzumab) + Endocrine Therapy (Letrozole, Anastrozole, Exemstane OR Fulvestrant)	Phase III *N* = 496 Multiple centers worldwide	Progression-free survival (PFS)	Metastatic
Tucatinib, Palbociclib, and Letrozole in Metastatic Hormone Receptor-Positive and HER2-positive Breast Cancer (*NCT03054363*) [83]	Tucatinib + Palbociclib + Letrozole	Phase Ib/II *N* = 42 USA	Phase 1b adverse events (AE) Progression-free survival (PFS)	Metastatic
Palbociclib and Trastuzumab With Endocrine Therapy in HER2-positive Metastatic Breast Cancer (PATRICIA II) (*NCT02448420*) [84]	Palbociclib + Trastuzumab (HR-/HER2+) Palbociclib + Trastuzumab (HR+/HER2+) Trastuzumab + Palbociclib + Letrozole (HR+/HER2+) Palbociclib, trastuzumab, and endocrine therapy (Aromatase Inhibitor, Fulvestrant, or Tamoxifen) for HR-positive, HER2 positive, Luminal intrinsic subtype (PAM50) Control arm: Physician’s choice (T-DM1 or chemotherapy [gemcitabine, vinorelbine, capecitabine, eribulin or taxane] + trastuzumab or endocrine therapy + trastuzumab) for HR-positive, HER2 positive, Luminal intrinsic subtype (PAM50)	Phase II *N* = 102 Spain	Progression-Free Survival (PFS)	Metastatic
Anastrozole, Palbociclib, Trastuzumab and Pertuzumab in HER2-positive, HER2-positive Metastatic Breast (*NCT03304080*)	Anastrozole + Palbociclib + Trastuzumab + Pertuzumab	Phase I/II *N* = 36 USA	Dose-Limiting Toxicity (DLT) Maximum Tolerated Dose (MTD) Clinical Benefit Rate (CBR)	Metastatic
T-DM1 With or Without Abemaciclib for the Treatment of HER2-Positive Metastatic Breast Cancer (*NCT04351230*)	Control arm: T-DM1 T-DM1 + Abemaciclib	Phase II *N* = 0 (Withdrawn) USA	Progression-free survival (PFS)	Metastatic
Pyrotinib, Letrozole and SHR6390 in ER+/HER2+ Advanced Breast Cancer (PLEASURABLE) (*NCT03772353*) [85]	Pyrotinib + Letrozole + Dalpiciclib (CDKi) (SHR6390) Pyrotinib + Fulvestrant + Dalpiciclib	Phase I/II *N* = 79 China	Phase 1b adverse events (AE) Progression-free survival (PFS)	Metastatic
Pyrotinib with CDKi SHR6390 for Trastuzumab-treated Advanced HER2-Positive Breast Cancer (INPHASE) (*NCT04095390*)	HR+/HER2+: Pyrotinib + Dalpiciclib + Letrozole HR-/HER2+: Pyrotinib + Dalpiciclib + Capecitabine HR-/HER2+: Pyrotinib + Dalpiciclib	Phase II *N* = 60 China	Objective Overall Response Rate (ORR)	Metastatic

** This table was prepared based on the information publicly available in the National Clinical Trials (NCT) registry and is subject to change (https://www.clinicaltrials.gov/, (accessed on 7 June 2022)).

## Abbreviations

BC: Breast cancer; CDKi: CDK 4/6 inhibitor; DoR: Duration of response; E2: Estrogen; EBC: Early breast cancer; ER: Estrogen receptor; HER2: Human epidermal growth factor receptor 2; HR: Hormonal receptor; MBC: Metastatic breast cancer; ORR: Objective response rate; pCR: Pathological complete response; PFS: Progression-free survival; PR: Progesterone receptor; RTK: Receptor tyrosine kinase.
